# Deep brain stimulation induces antiapoptotic and anti-inflammatory effects in epileptic rats

**DOI:** 10.1186/s12974-015-0384-7

**Published:** 2015-09-04

**Authors:** Beatriz O. Amorim, Luciene Covolan, Elenn Ferreira, José Geraldo Brito, Diego P. Nunes, David G. de Morais, José N. Nobrega, Antonio M. Rodrigues, Antonio Carlos G. deAlmeida, Clement Hamani

**Affiliations:** Disciplina de Neurofisiologia, Universidade Federal de São Paulo, Rua Botucatu, 862 5 andar, 04023-062 São Paulo, Brazil; Behavioural Neurobiology Laboratory, Centre for Addiction and Mental Health, Toronto, Canada; Departamento de Engenharia de Biossistemas, Universidade Federal de São João del-Rei, São João del-Rei, MG 36301-160 Brazil; Division of Neurosurgery, Toronto Western Hospital, University of Toronto, Toronto, Canada

**Keywords:** Anterior thalamic nucleus, Thalamus, Seizures, Deep brain stimulation, Epilepsy, Apoptosis, Neuroprotection, Caspase

## Abstract

**Background:**

Status epilepticus (SE) is a severe condition that may lead to hippocampal cell loss and epileptogenesis. Some of the mechanisms associated with SE-induced cell death are excitotoxicity, neuroinflammation, and apoptosis.

**Objective:**

The objective of the present study is to test the hypothesis that DBS has anti-inflammatory and antiapoptotic effects when applied during SE.

**Methods:**

Rats undergoing pilocarpine-induced SE were treated with anterior thalamic nucleus (AN) deep brain stimulation (DBS). Inflammatory changes and caspase 3 activity were measured within 1 week of treatment.

**Results:**

In pilocarpine-treated rats, DBS countered the significant increase in hippocampal caspase 3 activity and interleukin-6 (IL-6) levels that follows SE but had no effect on tumor necrosis factor α (TNFα).

**Conclusions:**

DBS has anti-inflammatory and antiapoptotic effects when given to animals undergoing status.

**Electronic supplementary material:**

The online version of this article (doi:10.1186/s12974-015-0384-7) contains supplementary material, which is available to authorized users.

Status epilepticus (SE) is a condition associated with continuous seizure activity that often leads to excitotoxicity and cell death [1]. Deep brain stimulation (DBS) of the anterior thalamic nucleus (AN), an approved treatment for medically refractory partial epilepsy [2], has been shown to reduce seizure rate and increase the latency for the development of SE in different rodent models [3–6]. To date, whether stimulation is protective against SE-induced excitotoxicity is largely unknown.

In Parkinson’s disease, subthalamic nucleus DBS has been proposed to induce neuroprotective effects by reducing the glutamatergic drive to the substantia nigra [7]. In a parallel scenario, AN stimulation inhibits the spontaneous activity of local neuronal populations and reduces the firing rate of dentate gyrus cells [8]. Following this line of reasoning, we hypothesize that a decreased drive to the hippocampus following AN DBS could have neuroprotective effects.

In preclinical models [9] and in the clinic [10, 11], neuromodulation treatments that induce antiepileptic effects, such as vagus nerve stimulation (VNS), have been shown to reduce plasmatic levels of cytokines. 

In the present study, we test the hypothesis that AN DBS reduces hippocampal apoptosis and neuroinflammation in rats undergoing pilocarpine (Pilo)-induced SE.

## Methods

### Ethics, consent, and permissions

Experiments were approved by the Animal Care committee of the Universidade Federal de São Paulo (1482/11).

### Pilocarpine administration and AN stimulation

Male Wistar rats (~250 g) were anesthetized with ketamine/xylazine (100/7.5 mg/kg i.p.) and had insulated stainless steel electrodes (cathodes; 250-μm diameter; 0.5-mm exposed surface) bilaterally implanted into the AN (anteroposterior −1.5, lateral ± 1.5, depth 5.2) [12]. A screw implanted over the right somatosensory cortex was used as the anode. Control animals had holes drilled into the skull but were not implanted with electrodes. Animals undergoing electroencephalography (EEG) recordings were implanted with bipolar cortical electrodes (Plastics One; 3 mm posterior, 4 mm lateral, and 2 mm ventral to the bregma) [12]. One week later, animals received *N*-methyl-scopolamine (1 mg/kg s.c. Sigma, St Louis, MO) followed, 30 min later, by pilocarpine (320 mg/kg, i.p., Vegeflora, Brazil). SE was characterized by the presence of uninterrupted behavioral seizures [24]. As pilocarpine-induced seizures often last a few seconds, DBS was commenced 1 minute after SE onset. Stimulation settings were in the range of those used in our previous studies: 130 Hz, 90 μs, and 400 μA (St Jude MTS, Plano, TX) [3].

Ninety minutes after SE, the animals received thionembutal (30 mg/kg) to attenuate behavioral status and reduce mortality rate. As animals began to recover 6 h later, DBS was delivered continuously during the first 6 h of status.

EEG was recorded with a BNT-36 system (Lynx, Brazil) using the ENSA software (ENSA, Brazil) for 24 h from the moment Pilo was injected. Signals were amplified, band pass filtered (0.1–30 Hz), and digitized (200 samples/s). Complex Gaussian’s wavelet transform analysis was used to investigate the signals in the frequency domain. Placement of DBS electrodes was confirmed in cresyl-violet-stained sections and was similar to that described in our previous reports [13].

### Caspase 3 and cytokine measurements

After removal from the skull, the brains were split in half, and the hippocampus of each hemisphere was dissected. The left hippocampus was used for the study of caspase activity. The right hippocampus was used to measure cytokines.

The hippocampus of each animal was incubated with a homogenization buffer and mechanically homogenized, as previously described [14]. Samples were centrifuged for 40 min, and the total protein content in the supernatant determined using the Bio-Rad Protein Assay, according to the manufacturer’s specification (Bio-Rad Labs, Germany). Measurement of caspase 3 activity was obtained using the caspase 3 fluorimetric assay kit (CASP3F, Sigma, USA). Triplicates of each sample (all containing 100 mg of protein) were analyzed. All received the substrate but only one the inhibitor. Activity was measured continuously over 5 h on a FlexStation 3 Spectrofluorimeter (Molecular Probes, USA), using λex = 360 nm and λem = 465 nm. Values obtained from samples containing the inhibitor were subtracted from those recorded from the other samples, and the arithmetic average was calculated. After calibration for AMC, results were expressed as nMol AMC.

For cytokine detection, the right hippocampus was homogenized in 0.01 M Tris hydrochloride (pH 7.6) containing 5.8 % sodium chloride, 10 % glycerol, 1 % Nonidet P40 (NP-40), 0.4 % of ethylenediamine tetraacetic acid (EDTA), and protease inhibitors. Samples were sonicated and stored at −80 °C. Subsequently, samples were centrifuged for 5 min. at 10,000 × *g* at 4 °C, and concentrations determined with a Millipore multiplex Rat Cytokine Kit (RECYTMAG-65K-03) on the Luminex® xMAP® platform (xPonent/Analyst Software version 4.2). Longitudinal controls were used to assess inter-assay variability. Results are expressed in pg/mg.

### Statistical analysis

Two-way ANOVA (DBS and Pilo as main factors; Tukey post hoc) was used to compare neurochemical data. Electrophysiological results were analyzed with Wilcoxon signed-rank test. Values in the text represent mean ± SEM.

## Results

### Apoptosis and neuroinflammatory changes

**Fig. 1:**
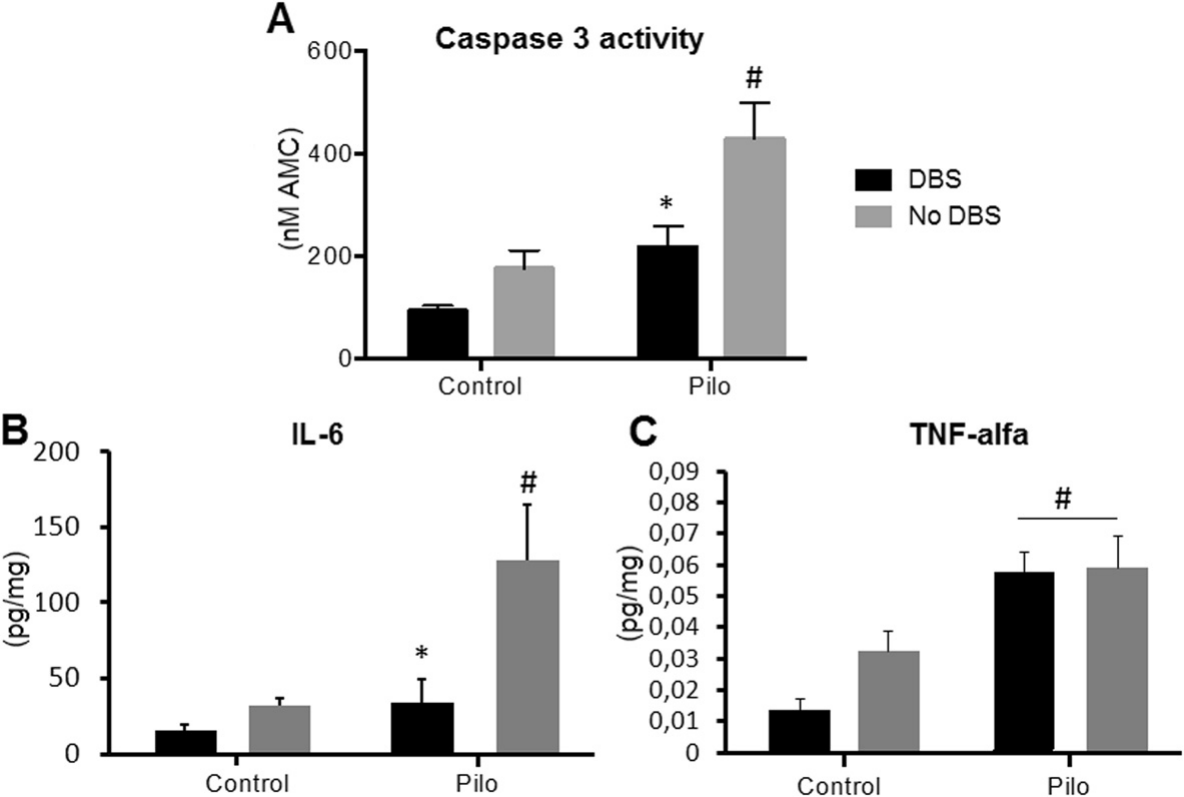
DBS, apoptosis, and neuroinflammation. (**a**) Hippocampal caspase 3 activity was increased in pilocarpine-treated rats undergoing SE (*P* = 0.009; vs saline controls), an effect that was significantly reversed in the DBS-treated Pilo group (*P* = 0.02; vs Pilo). Similarly (**b**), hippocampal levels of the pro-inflammatory IL-6 were significantly increased after SE (*P* = 0.006; vs saline-injected controls), an effect that was countered by the administration of AN DBS (*P* = 0.02; vs Pilo). In contrast, SE-induced increases in TNFα (*P* = 0.0003 vs saline-injected controls) were not influenced by DBS (**c**). # indicates differences from *control*, *indicates differences from *Pilo*

Hippocampal caspase 3 activity was studied 7 days following SE, a time frame during which apoptosis reaches maximal levels [15, 16]. At this time point, we found significant Pilo (*F*_(1,16)_ = 17.17; *P* = 0.0008) and DBS effects (*F*_(1,16)_ = 10.34; *P* = 0.005). As can be seen in Fig. 1, caspase 3 activity was increased in pilocarpine-treated rats (*n* = 5) as compared to saline-injected controls (*n* = 5; *P* = 0.009). The administration of DBS significantly reduced such activity (*n* = 5), bringing it to a level that was similar to that of non-epileptic controls (*n* = 5; *P* = 0.02 vs Pilo). In contrast, no significant Pilo (*F*_(1,16)_ = 0.01; *P* = 0.95) or DBS effects (*F*_(1,16)_ = 1.97; *P* = 0.18) were recorded when caspase 3 activity was measured 24 h following status epilepticus (Additional file 1: Figure S1; *n* = 5 animals/group).

Markers of inflammatory activity were studied 1 day following status, when post-SE changes are at high levels [17, 18]. Hippocampal pro-inflammatory IL-6 was influenced by both Pilo (F_(1,14)_ = 9.85; *P* = 0.007) and DBS (F_(1,14)_ = 9.23; *P* = 0.008) with interaction between both factors (F_(1,14)_ = 4.53; *P* = 0.05). Overall, levels of this interleukin were slightly reduced in AN DBS control animals, a response that was further evidenced after status (*P* = 0.002 vs Pilo) (Fig. 1). Although TNFα levels were in the lower limit of the detection of the fluorimetric assay, these were found to be influenced by Pilo (F_(1,14)_ = 22.60; *P* = 0.0003) but not DBS.

To investigate whether the protective effects of DBS were due to a decrease in SE severity, EEG recordings were obtained before and during status (*n* = 5 per group). Though tracings looked similar between groups, spectral analysis revealed that animals receiving DBS had a significant decrease in alpha and beta band peaks (Additional file 2: Figure S2). This suggests that DBS may potentially decrease SE severity.

## Discussion

It is well established that seizures and SE can activate intrinsic and extrinsic apoptotic pathways leading to neuronal death. As a common final step, caspase 3 activation invariably leads to an irreversible apoptotic process. In rodents, caspase 3 is active 24–72 h following status but maximally expressed 7 days later [19]. Our results suggest that animals given DBS during status had a significant decrease in hippocampal caspase 3 activity, as measured by a commercial protein assay. While this method was reliable and showed low variability across animals, it did not allow us to determine in which hippocampal subregions apoptosis was occurring. In a preliminary immunohistochemistry experiment (unpublished data), we found that animals undergoing SE with or without DBS had caspase-3-expressing cells in the dentate gyrus, CA1, and CA3 subfields. This technique, however, has only shown sparsely stained cells and a great variability across rats. Whether the effects of DBS are related to a decrease in apoptotic processes or a lower number of hippocampal cells undergoing cell death remains to be elucidated.

Another technical aspect that needs to be discussed is the choice of a control group without electrodes implanted. This was based on our previous studies showing that electrode insertion did not influence the latency for developing SE or the frequency of seizures in pilocarpine-treated rats [3, 20]. Placement of electrodes in the brain often causes a mild local inflammatory response that tends to subside over time. Though it is possible that this might have occurred in the AN, samples in our study were collected at a distance from the target (i.e., in the hippocampus and not in the AN). In addition, an inflammatory response should have theoretically increased pro-inflammatory cytokines. In our study, DBS-treated animals had the opposite effect.

Most cells in the AN are immunocytochemically positive for glutamate and aspartate [21]. Connections between the AN and hippocampus are both direct (e.g., via subiculum and CA1) and indirect (i.e., via anterior cingulum and entorhinal cortex) [22, 23]. In our previous work, AN DBS was shown to induce a strong depolarization block of local neuronal populations and reduce the firing in hippocampal dentate gyrus cells [24]. Bearing this in mind, it is possible that reduced activity within the AN complex and DG following DBS might have decreased glutamate-induced excitotoxicity, reducing hippocampal caspase activity and inflammatory responses. This, however, still needs to be demonstrated.

Another possibility to explain potential neurochemical differences between animals that did or did not receive DBS is that stimulation might have influenced SE severity. Though EEG tracings looked similar between groups, a more detailed spectral analysis revealed that the rats given DBS had a decrease in alpha and beta power. This may represent a milder form of status epilepticus, as the withdrawal of anticonvulsant medications may enhance beta rhythms during continuous seizures [25].

One limitation of the current study is that we cannot infer a direct association between reduced excitotoxicity, anti-inflammatory, and antiapoptotic effects of DBS. Similarly, we are unable to explain why DBS has only influenced certain pro-inflammatory cytokines (i.e., IL-6 but not TNFα).

## Conclusion

As excitotoxicity and inflammatory responses are early occurring events following SE, we postulate that the reduced levels of apoptosis observed after DBS may be due, at least in part, to its anti-inflammatory effects. That said, our results do not allow one to establish a causal relationship between a DBS-induced reduction in cytokines and apoptosis, an aspect that needs to be investigated in the future.

## Additional files

Additional file 1: Figure S1. Apoptosis 24 h after pilocarpine-induced status epilepticus. No significant differences were found in hippocampal caspase 3 activity when animals who developed pilocarpine-induced SE with or without DBS were compared to controls. (TIFF 94 kb) Additional file 2: Figure S2. EEG activity recorded before and during SE in animals that did and did not receive DBS. (A) Traces of a non-stimulated animal before (left) and during (right) SE. Spectral arrays after wavelet transform of corresponding EEG traces show activity in the α and β bands during SE. (B) Traces of a DBS-treated animal before (left) and during (right) SE. Spectral arrays after wavelet transform of the corresponding EEG traces show that activity during SE in the α and β bands are reduced during AN DBS. (C) Box plot showing the mean power spectrum of different frequency bands pre-SE in animals that did not (yellow bars) or did receive AN DBS (blue bars). As stimulation was not turned on at that point, Wilcoxon signed-rank test showed no significant difference between groups (*P* > 0.05, *n* = 5). (D) Box plot showing the mean power spectrum of different frequency bands during SE without (yellow bars) and with AN DBS (blue bars). Wilcoxon signed-rank test showed significant differences between groups in the α and β bands (*P* < 0.05, *n* = 5 animals/group). (TIFF 2135 kb)
