# Supplementary figures and images for: Deep brain stimulation induces antiapoptotic and anti-inflammatory effects in epileptic rats
Source: J Neuroinflammation. 2015 Sep 4;12:162. doi: 10.1186/s12974-015-0384-7 (PMC4558969; doi:10.1186/s12974-015-0384-7)

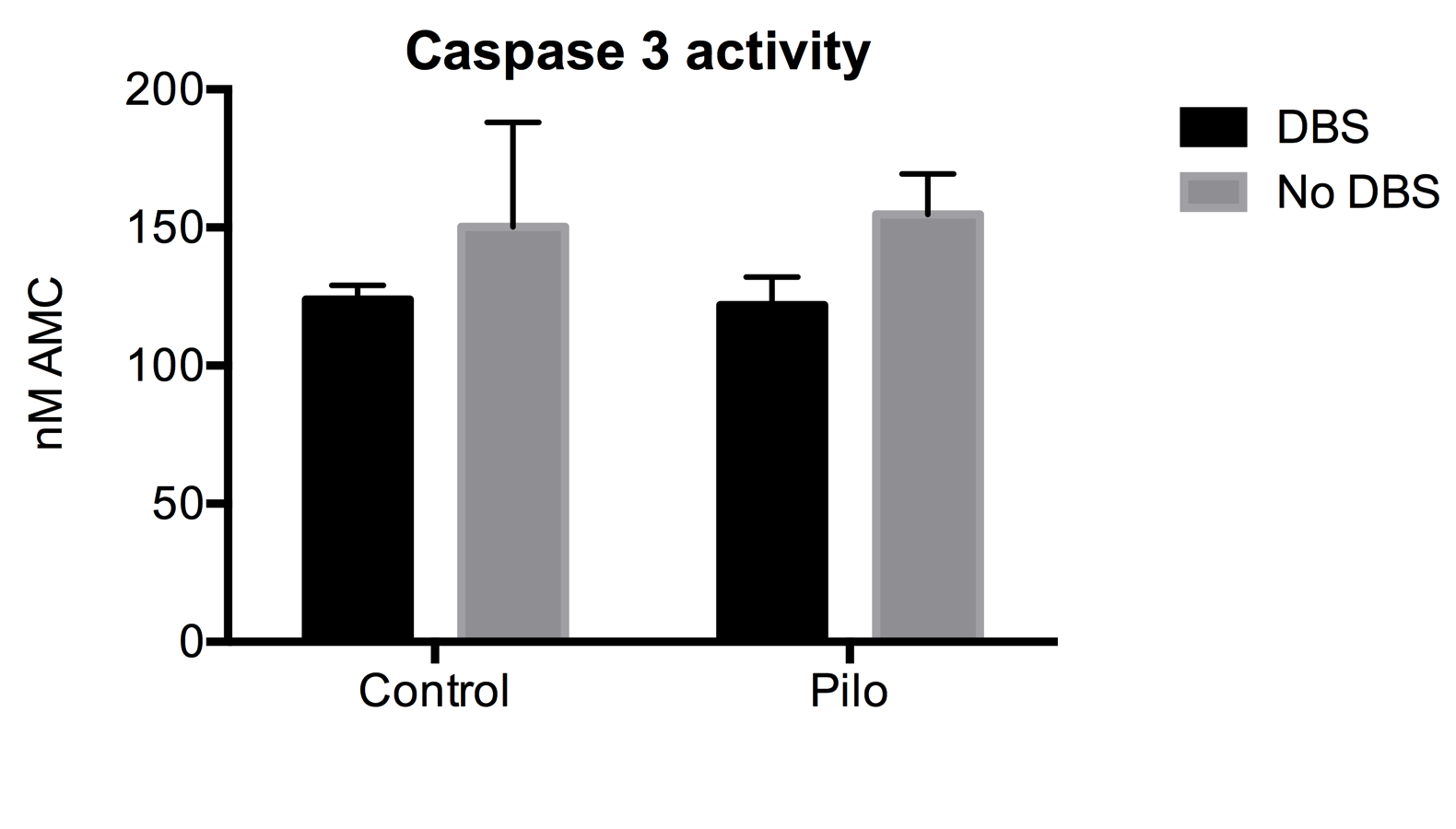

Supplement: Additional file 1: Figure S1. — Apoptosis 24 h after pilocarpine-induced status epilepticus. No significant differences were found in hippocampal caspase 3 activity when animals who developed pilocarpine-induced SE with or without DBS were compared to controls. (TIFF 94 kb) [file 12974_2015_384_MOESM1_ESM.tiff]

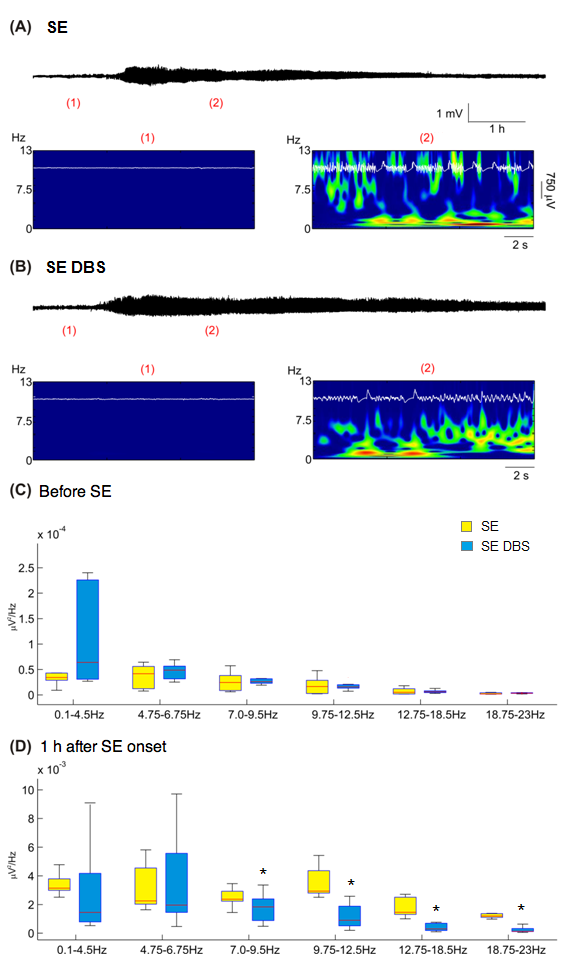

Supplement: Additional file 2: Figure S2. — EEG activity recorded before and during SE in animals that did and did not receive DBS. (A) Traces of a non-stimulated animal before (left) and during (right) SE. Spectral arrays after wavelet transform of corresponding EEG traces show activity in the α and β bands during SE. (B) Traces of a DBS-treated animal before (left) and during (right) SE. Spectral arrays after wavelet transform of the corresponding EEG traces show that activity during SE in the α and β bands are reduced during AN DBS. (C) Box plot showing the mean power spectrum of different frequency bands pre-SE in animals that did not (yellow bars) or did receive AN DBS (blue bars). As stimulation was not turned on at that point, Wilcoxon signed-rank test showed no significant difference between groups (P > 0.05, n = 5). (D) Box plot showing the mean power spectrum of different frequency bands during SE without (yellow bars) and with AN DBS (blue bars). Wilcoxon signed-rank test showed significant differences between groups in the α and β bands (P < 0.05, n = 5 animals/group). (TIFF 2135 kb) [file 12974_2015_384_MOESM2_ESM.tiff]
